# Views on Ageing and Loneliness in Older Patients Undergoing Geriatric Treatment

**DOI:** 10.3390/geriatrics11050125

**Published:** 2026-09-07

**Authors:** Luise Umfermann, Aline Schönenberg, Tino Prell

**Affiliations:** 1 Department of Geriatrics, University Hospital Halle, 06120 Halle (Saale), Germany; 2 Department of Geriatrics, Jena University Hospital, 07743 Jena, Germany

**Keywords:** loneliness, views on ageing, subjective age, geriatric patients, depressive symptoms, geriatric assessment, rehabilitation

## Abstract

**Background/Objectives**: Loneliness is a clinically relevant psychosocial factor in later life and has been associated with adverse mental, cognitive, and physical health outcomes. Views on ageing may shape social engagement, coping, and perceived belonging, but evidence from geriatric patient populations remains limited. This study examined whether views on ageing are associated with loneliness in geriatric patients. Secondary analyses explored subjective age, including felt age and the perceived onset of old age. **Methods**: This cross-sectional analysis used baseline data from two prospective observational studies in geriatric patients (N = 459). Loneliness was assessed with the three-item UCLA Loneliness Scale. Views on ageing were measured using five items from the German Ageing Survey. Associations were examined using Spearman correlations, non-parametric group comparisons, and multiple linear regression. **Results**: Participants had a mean age of 83.1 years and 65.6% were female. The mean UCLA loneliness score was 1.54 ± 2.07, and the mean views on ageing sum score was 13.21 ± 2.72. More negative views on ageing were associated with higher loneliness (ρ = 0.122, *p* = 0.009). In the fully adjusted regression model, views on ageing remained independently associated with loneliness after adjustment for chronological age, sex, and depressive symptoms (β = 0.117, *p* = 0.014; adjusted R^2^ = 0.051). Depressive symptoms were also independently associated with loneliness (β = 0.182, *p* < 0.001), whereas chronological age and sex were not. At the item level, associations with loneliness were mainly driven by reduced positive ageing-related beliefs. Higher felt age was associated with higher loneliness (ρ = 0.151, *p* = 0.003), while a later perceived onset of old age was associated with lower loneliness (ρ = −0.136, *p* = 0.017). **Conclusions**: In geriatric patients, more negative views on ageing were weakly associated with higher loneliness independent of depressive symptoms. The small effect size suggests that views on ageing should be interpreted as one modest component within a broader biopsychosocial understanding of loneliness in geriatric care.

## 1. Introduction

Loneliness is increasingly recognized as a clinically relevant psychosocial factor in later life. It is commonly defined as the subjective experience of a discrepancy between desired and actual social relationships, and should be distinguished from objective social isolation, which refers to the structural availability or frequency of social contacts [1]. In older adults, loneliness has been associated with adverse health outcomes, including poorer cardiovascular health, increased depressive symptoms, suicidal ideation, cognitive decline, dementia risk, and mortality [2,3,4,5]. These associations are particularly relevant in geriatric patients, who often experience functional limitations, multimorbidity, reduced mobility, and transitions in living arrangements, all of which may increase vulnerability to perceived social disconnection [6,7].

Views on ageing (VoA) represent another important psychosocial dimension of later life. They comprise individual beliefs, expectations, and evaluations concerning one’s own ageing as well as more general age-related stereotypes. Such views are not merely descriptive attitudes, but have been linked to cognitive, mental, functional, and somatic health outcomes across adulthood and old age [8,9,10]. Positive self-perceptions of ageing have been associated with better cognitive functioning [11,12], lower dementia-related risk indicators [13,14], lower cardiovascular risk [15], and even longer survival [5]. These findings suggest that views on ageing may influence health and behaviour through motivational, affective, and behavioural pathways.

Conceptually, views on ageing may also be relevant for loneliness. Negative age stereotypes and an older subjective age may promote withdrawal, reduced social initiative, and lower expectations regarding participation and social belonging. Conversely, positive views on ageing may support adaptive coping, maintenance of social roles, and continued engagement despite functional limitations. This interpretation is consistent with stereotype embodiment theory, which proposes that age stereotypes internalized across the life course can become self-relevant in later life and influence psychological, behavioural, and physiological processes [16]. It also aligns with the model of selection, optimization and compensation, according to which successful adaptation in later life depends on selecting meaningful goals, optimizing available resources, and compensating for losses [17,18].

Empirical studies have begun to examine the relationship between subjective ageing and loneliness. In adults aged 50 years and older, subjective age has been shown to shape the association between close social relationships and loneliness [19]. Other studies reported links between loneliness, depressive symptoms, subjective life expectancy, and felt age [20,21]. In older Chinese adults, resilience and self-esteem mediated the association between subjective age and loneliness [22]. Moreover, loneliness has been identified as a mediator between internalized negative age stereotypes and anxiety–depressive symptoms in older adults who perceived themselves as old [23]. A recent Chilean study further showed that negative self-perceptions of ageing were associated with loneliness in adults over 50 years, suggesting that this relationship may be observable across different cultural and social contexts [24].

However, several gaps remain. First, previous studies have mainly focused on community-dwelling or population-based samples, whereas geriatric patients with multimorbidity, functional limitations, and acute or post-acute care needs are underrepresented. Second, subjective ageing has often been operationalized primarily through felt age, while broader views on ageing, including positive and negative expectations and experiences of ageing, have been less consistently examined. Third, depressive symptoms are highly relevant in this context. Depression is common in older adults, frequently underrecognized, and closely related to both loneliness and negative perceptions of ageing [25,26,27,28]. It therefore remains important to determine whether the association between views on ageing and loneliness persists independently of depressive symptoms.

## 2. Materials and Methods

### 2.1. Study Design and Participants

This cross-sectional analysis used baseline data from two prospective observational studies on self-management and psychosocial factors in geriatric patients in Germany: the SelfManGer study (German Clinical Trials Register: DRKS00031016) and the JenaGer study (German Clinical Trials Register: DRKS00032328). Data were collected between February 2023 and August 2024 at geriatric wards in Saxony-Anhalt and Thuringia, Germany. The data and study protocols have been described elsewhere [7,29]. The study was conducted in accordance with the Declaration of Helsinki, and approved by the Ethics Committee of the University Hospital Halle and the State Chamber of Physicians of Saxony-Anhalt (Landesärztekammer) (protocol code 2022-026; 24 May 2022) and by the Ethics Committee of the University Hospital Jena (protocol code 2023-2923-BO).

These studies included hospitalized geriatric patients treated within the geriatric early rehabilitative complex treatment according to the German Operations and Procedures Key system (OPS 8-550), a multidisciplinary treatment approach (nurses, occupational and physical therapists, social workers, medical doctors, psychologists) spanning 14 to 21 days after acute illness. Patients were excluded if they were unable to provide valid self-report due to delirium, severe dementia, severe medical instability, vigilance disorders, severe hearing or speech problems, language barriers, or other medical reasons precluding participation. Written informed consent was obtained from all participants. The studies were approved by the responsible local ethics committees.

Variables extracted for the present analysis included age, sex, marital status, living situation, education, depressive symptoms, and functional independence in basic activities of daily living. Complete cases for loneliness and views of ageing were available for N = 459 participants, serving as the main study sample.

### 2.2. Variables of Interest

#### 2.2.1. Loneliness

Loneliness was assessed using the three-item UCLA Loneliness Scale [30,31]. Participants rated three statements: “I lack companionship”, “I feel left out”, and “I feel isolated from others”. Responses were given on a four-point Likert scale ranging from strong disagreement to strong agreement. The three items were summed to generate a total loneliness score, with higher values indicating greater loneliness. The UCLA Loneliness Scale shows strong test–retest reliability (r = 0.73) and good internal consistency (α 0.89–0.94) [32].

#### 2.2.2. Views on Ageing

Views on ageing (VoA) were assessed using five items from the German Ageing Survey (DEAS), a representative, nationwide biennial survey conducted with older adults in Germany [33,34]. The DEAS covers a broad range of validated psychosocial and health-related instruments, and thus serves as a reference point for many further studies conducted with older adults in Germany. Items covered negative and positive evaluations of ageing, including perceived deterioration with age, vitality, usefulness, expectations regarding ageing, and happiness compared with younger age. Responses were given on a four-point Likert scale. Items were recoded where necessary so that higher item values consistently indicated more negative views on ageing. A sum score was calculated across the five items. The items selected for the DEAS were selected from the Philadelphia Geriatric Center Morale Scale [35] and applied in several nationwide survey waves from 1996 to 2025. Detailed information on the DEAS and its instrument can be found in the respective documentation [33,34].

#### 2.2.3. Subjective Age

The ageing process is highly complex and can be represented using various cognitions and measurement approaches [10,36]. While VoA reflect one measure of expectations regarding the ageing process, a multi-variate assessment of ageing is recommended to capture its multidimensionality. Thus, to understand ageing perception in more depth and to control for the individual relevance of ageing cognition, subjective age was assessed with two further variables in addition to the VoA sum score. First, participants were asked to indicate their felt age in years: “Irrespective of your actual age, how old do you feel?” Felt age was analysed as a continuous variable. For descriptive and group-based analyses, felt age was additionally categorized relative to chronological age as feeling younger, the same age, or older than one’s actual age.

Second, participants were asked to indicate the age from which they would consider a person to be “old”: “Starting from which age would you consider someone old?” This variable was used as a continuous covariate of the perceived onset of old age. Higher values indicate that old age is perceived to begin later.

Chronological age in years was used as a sociodemographic covariate in all adjusted regression models. Thus, analyses of felt age and perceived onset of old age were adjusted for actual age to distinguish subjective ageing perceptions from chronological age.

#### 2.2.4. Covariates

In order to describe the participating patients and to include relevant psychosocial and health-related covariates, the following variables were collected from routine data as well as via questionnaires.

- Gender (male/female).
- Marital status (single/married or with partner/widowed or separated).
- Living arrangement (alone/with partner or family/other).
- Education according to the German education system (low = 8 years or less, medium = 10 years or less, high = at least 12 years).
- Depressive symptomology was assessed using the Geriatric Depression Scale (GDS), a 15-item questionnaire using dichotomous items. It cumulates in a sum score, with higher scores indicating higher depressiveness, with a score of 5≥ indicating clinically relevant depressive symptomology [37,38].
- Functional health and activities of daily living were assessed using the Barthel Index, a 10-item expert-rated instrument assessing functional domains such as bathing, clothing, walking and preparing food. It ranges between 0–100 points, with higher scores indicating better functionality [39].

### 2.3. Statistical Analysis

Descriptive statistics were used to characterize the study sample. Continuous variables are reported as means and standard deviations or medians where appropriate. Categorical variables are reported as absolute and relative frequencies.

Associations between loneliness, views on ageing, depressive symptoms (GDS), and continuous or ordinal covariates were examined using Spearman’s rank correlation coefficients. Group differences were analysed using Kruskal–Wallis tests with Dunn’s post-hoc tests where appropriate. These analyses were used to describe bivariate associations and to identify relevant sociodemographic patterns.

Multiple linear regression models were then used to examine the association between views on ageing and loneliness. Loneliness was entered as the dependent variable. In the first model, views on ageing were entered as the main independent variable. In the second model, age and sex were added. In the third model, depressive symptoms (GDS) were added to examine whether the association between views on ageing and loneliness remained independent of depressive symptom burden.

Statistical analyses were performed using SPSS (version 30.0.0.0). A two-sided *p*-value below 0.05 was considered statistically significant. Analyses were conducted as complete-case analyses for the variables included in each model.

## 3. Results

### 3.1. Sample Characteristics

The analytical sample included geriatric patients aged 67 to 96 years. The mean age was 83.0 years, and approximately two-thirds of participants were female. More than half of the participants were widowed or separated, and about half lived alone. Educational attainment was almost equally distributed between medium and high education, whereas low educational attainment was uncommon in this cohort.

The mean loneliness score was low, although there was substantial variability between participants. The mean VoA sum score indicated a moderate level of negative views on ageing. Clinically relevant depressive symptom burden, defined as a GDS score of 6 or higher, was present in slightly more than one quarter of the cohort. Regarding subjective age, the mean felt age was lower than the mean chronological age, and the mean age from which participants considered a person to be “old” was approximately 77 years. Sample characteristics are given in Table 1.

### 3.2. Views on Ageing and Loneliness

More negative views on ageing were associated with higher loneliness, although the association was small in magnitude (ρ = 0.12, *p* = 0.01). At item level, the association with loneliness appeared to be driven mainly by items reflecting the absence of positive ageing-related beliefs. Specifically, loneliness was weakly associated with reduced vitality, less favourable expectations regarding ageing, and lower happiness compared with younger age. By contrast, the two items reflecting explicit negative age-related beliefs were not significantly associated with loneliness. Detailed item-level associations are shown in Supplementary Table S1.

### 3.3. Subjective Age and Loneliness

In addition to the VoA sum score, two further variables on subjective age were examined: felt age and the perceived age threshold from which a person is considered old. Higher felt age was associated with higher loneliness (ρ = 0.15, *p* < 0.01). In categorical analyses, participants who felt younger than their chronological age reported lower loneliness than those who felt the same age or older; the overall group difference was statistically significant (Kruskal–Wallis test, *p* < 0.01). Post-hoc comparisons indicated significant differences between participants feeling younger and those feeling the same age (*p* = 0.03), and between participants feeling younger and those feeling older (*p* = 0.01), whereas participants feeling the same age and those feeling older did not differ significantly (*p* = 0.83). Detailed bivariate associations and post-hoc comparisons are reported in Supplementary Tables S2 and S3.

### 3.4. Depressive Symptoms

Depressive symptoms were associated with loneliness (ρ = 0.16, *p* < 0.01) and VoA sum score (ρ = 0.27, *p* < 0.01). As observed for loneliness, the association between depressive symptoms and VoA was mainly related to the absence of positive ageing-related beliefs rather than to explicit negative ageing stereotypes. Higher GDS scores were also associated with higher felt age, whereas the perceived age threshold for being old was not associated with depressive symptoms. Detailed associations of depressive symptoms and covariates and post-hoc group comparisons are provided in Supplementary Tables S2 and S3.

### 3.5. Regression Models

In the unadjusted model, more negative views on ageing were associated with higher loneliness scores. This association remained significant after adjustment for chronological age and sex. After additional adjustment for depressive symptoms, the association was attenuated but remained statistically significant. Depressive symptoms were independently associated with loneliness, whereas age and sex were not significant predictors, neither were martial state, education or living situation (all *p* > 0.005). The fully adjusted model explained 5.9% of the variance in loneliness (Table 2).

Additional regression models were calculated for the two variables on subjective age. Felt age remained significantly associated with loneliness after adjustment for chronological age, sex, and depressive symptoms. Similarly, the age threshold from which someone was considered old remained inversely associated with loneliness in the fully adjusted model (Table 3).

## 4. Discussion

### 4.1. Principal Findings

In this cross-sectional study of geriatric patients, more negative views on ageing were associated with higher loneliness. The association was statistically significant, but small in magnitude. Importantly, it remained present after adjustment for chronological age, sex, and depressive symptoms, suggesting that the relationship between views on ageing and loneliness was not fully explained by depressive symptom burden. However, the proportion of explained variance was low, and the findings should therefore be interpreted with caution.

In addition to the overall views on ageing score, subjective age measured as felt age and the age at which someone is considered old were related to loneliness. Higher felt age was associated with higher loneliness, while a later perceived onset of old age was associated with lower loneliness. These findings suggest that loneliness in geriatric patients may be related not only to explicit evaluations of ageing, but also to how old individuals feel and where they locate the boundary of old age. However, these associations were also small and should be regarded as exploratory.

### 4.2. Interpretation of the Weak Association Between Views on Ageing and Loneliness

The weak association between views on ageing and loneliness may have several explanations. First, loneliness is a multifactorial phenomenon. It reflects the subjective discrepancy between desired and actual social relationships and is therefore not reducible to objective social isolation alone [1]. In older adults, loneliness has been associated with social network characteristics, bereavement, living arrangements, health status, functional impairment, depression, and broader psychosocial resources [5,40,41,42,43]. Views on ageing may therefore contribute to loneliness, but they are unlikely to be a dominant explanatory factor on their own.

Second, the study population consisted of geriatric patients with acute or post-acute care needs [6]. These patients were recruited in the context of geriatric early rehabilitative complex treatment and were characterized by multimorbidity, functional impairment, and clinical vulnerability. In such a population, immediate health problems, hospitalization, dependency, pain, mobility loss, or uncertainty about recovery may have a stronger influence on loneliness than general beliefs about ageing [44]. This may explain why the views on the ageing–loneliness association were detectable but small. The clinical context may therefore attenuate the role of broader psychosocial attitudes compared with more immediate health-related and functional determinants [44,45].

Third, loneliness was relatively low on average in this cohort. This distribution may have reduced the ability to detect stronger associations. A floor effect is plausible, particularly if many patients did not report loneliness despite functional limitations. In older clinical populations, loneliness may also be underreported due to stigma, social desirability, or the perception that loneliness is not a medical issue.

### 4.3. Role of the Views on Ageing Items

The item-level findings are particularly relevant for interpretation. The association with loneliness was not driven by the explicitly negative items: “As I get older, everything gets worse” and “As I get older, I am less useful.” Instead, the significant associations were found for items reflecting reduced positive ageing-related beliefs: “I have as much pep as last year,” “As I get older, things are better than I thought they would be,” and “I am as happy now as when I was younger.”

This pattern suggests that loneliness in geriatric patients may be more closely related to a lack of positive ageing-related expectations and experiences than to the explicit endorsement of negative age stereotypes. In other words, the absence of optimism, vitality, and continuity of happiness may be more relevant than strong agreement with negative beliefs about decline or uselessness. This interpretation is consistent with studies showing that positive self-perceptions of ageing may be particularly relevant for health and functioning in older age [5,8,16]. It also resonates with findings that reported that positive, but not negative, self-perceptions of ageing predicted cognitive function among older adults [11].

The item-level pattern may also reflect the specific situation of geriatric patients. In the context of acute illness or functional decline, patients may not necessarily endorse global negative stereotypes such as being less useful or perceiving everything as worse with age. However, they may experience a concrete loss of energy, vitality, and positive future expectations. These aspects may be more proximally related to loneliness because they affect motivation, perceived participation, and social engagement.

This interpretation is further supported by the finding that depressive symptoms were associated mainly with the same positively framed views on ageing items. Depressive symptoms may reduce perceived vitality, positive expectations, and retrospective comparison with younger age. Therefore, the overlap between depressive symptoms, loneliness, and less positive views on ageing may reflect a shared affective component. Nevertheless, views on the ageing–loneliness association remained significant after adjustment for GDS, suggesting that the association cannot be attributed solely to depressive symptoms.

### 4.4. Subjective Age

The findings for felt age and perceived onset of old age extend the results for the views on ageing sum score. Higher felt age was associated with higher loneliness, even after adjustment for chronological age, sex, and depressive symptoms. This is consistent with previous studies indicating that subjective age is linked to loneliness and related psychosocial outcomes [19,21,22] further reported associations between subjective age trajectories, loneliness, and stress across adulthood, although effects differed by age group.

Felt age may capture aspects of vulnerability, reduced vitality, or social disengagement that are not fully reflected by chronological age. Conversely, participants who located the onset of old age at a later age reported lower loneliness. This may indicate that a broader or more flexible perception of ageing is associated with lower social disconnection. Such a finding is compatible with theoretical perspectives suggesting that age stereotypes and subjective ageing experiences can become self-relevant and shape behaviour, affect, and social participation [8,16].

However, these findings should not be overinterpreted. Felt age and age threshold are single items and may reflect multiple constructs, including health status, functional capacity, personality, cultural norms, and current mood. They should therefore be interpreted as exploratory markers of subjective ageing rather than as robust explanatory mechanisms.

Depressive symptoms were associated with both loneliness and more negative views on ageing. In the regression model, depressive symptoms were independently associated with loneliness, while the association between views on ageing and loneliness was attenuated but remained significant. This implies that a part of the association between views on ageing and loneliness may be attributed to changes in mood, however, due to the cross-sectional nature of the data, this effect cannot be interpreted causally. Still this finding is consistent with previous research showing close links between depressive symptoms and loneliness in older adults [25,26], indeed, loneliness is a key risk factor for developing depression [46]. Depression is also common in later life and may be underrecognized in routine care, particularly in older patients with somatic comorbidity and functional impairment [27,28]. A recent review shows that positive attitudes towards ageing were associated with less, and negative attitudes towards ageing with more depressive symptoms [47], and that attitudes toward ageing further influences hopefulness. These findings overall imply a complex interplay of social embeddedness, ageing expectations and mood that should be assessed in detail in future research.

### 4.5. Clinical Implications

The clinical relevance of the findings lies less in the size of the statistical association and more in the psychosocial perspective they support. Loneliness in geriatric patients should not be understood solely as a consequence of living alone or lacking social contacts. This becomes evident in the fact that sociodemographic information was not statistically significant when considering views on ageing and depressive symptoms. Instead, loneliness may also be related to how patients experience their own ageing, vitality, usefulness within society, and future expectations. Social roles oftentimes change with advancing age, especially after retirement and upon restrictions in activities due to health; finding purpose via new social roles and activities may combat loneliness above and beyond mere social contact [48,49]. Meaningful social contact, either on a community-level (e.g., community-meals or activities) or via volunteering, may give older adults purpose within society [50]. A recent meta-analysis shows clear associations between purpose in life and depressiveness [51], yet these aspects are rarely assessed systematically in geriatric care. Future research should aim to understand how social contact, societal roles, purpose in life and views on ageing contribute to mood and loneliness in older age in order to derive at meaningful intervention programs.

At the same time, the weak effect size argues against overemphasizing views on ageing as a primary target for loneliness interventions. Rather, views on ageing may be one component within a broader biopsychosocial assessment. Interventions aiming to reduce loneliness in geriatric patients should primarily address social connectedness, functional recovery, mobility, depressive symptoms, and environmental barriers. Positive views on ageing may be considered an additional resource-oriented target, for example through psychoeducation, strengths-based communication, activation, and rehabilitation goals that emphasize capability and participation. This is consistent with the model of selection, optimization, and compensation, which emphasizes adaptive goal selection, resource optimization, and compensation for losses in later life [17].

### 4.6. Limitations

Several limitations need to be considered. First, the cross-sectional design does not allow causal conclusions. It remains unclear whether more negative views on ageing contribute to loneliness, whether loneliness shapes the perception of ageing, or whether both are influenced by common underlying factors such as functional impairment, depressive symptoms, or poor health. Second, the cohort consisted of geriatric patients in acute or post-acute clinical care. This is a strength in terms of clinical relevance, because geriatric patients are underrepresented in previous research on views on ageing and loneliness. However, it also limits generalizability to healthier community-dwelling older adults. The hospital context may have influenced responses to subjective questions, particularly those concerning ageing, vitality, and loneliness.

Third, both loneliness and views on ageing were assessed by self-report. Social desirability and stigma may have led to underreporting of loneliness. Similarly, responses to views on ageing items may have been influenced by current illness, mood, rehabilitation expectations, or acute functional loss.

Fourth, the views on ageing scale used in this study contained only five items. While these items capture relevant aspects of ageing-related beliefs and experiences, they do not fully represent the multidimensionality of views on ageing. The item-level pattern suggests that positive and negative views on ageing may not simply be opposite poles of a single construct. Future studies should consider more differentiated views on ageing instruments that distinguish between physical decline, social loss, continuous growth, and self-related gains, as proposed in multidimensional approaches to subjective ageing [8].

Fifth, the explained variance in the regression models was low. This indicates that most variance in loneliness remained unexplained by views on ageing, age, sex, and depressive symptoms. Future studies should include more comprehensive social, functional, and environmental variables, such as social network quality, bereavement, sensory impairment, mobility, participation, perceived social support, and discharge destination. Especially social engagement and relations prior to hospitalization may serve as fruitful explanatory variables and should be considered in future research. Notably, sociodemographic information such as living arrangements and marital status did not have an impact on loneliness when other variables such as VOA and depressive symptoms were considered, indicating that loneliness goes beyond mere social contact.

## 5. Conclusions

The present findings suggest that subjective views on ageing constitute one independent, albeit modest, psychosocial factor associated with loneliness in geriatric patients. Rather than indicating that loneliness is primarily linked to explicit negative age stereotypes, the results point to the potential importance of maintaining positive perceptions of ageing. At the same time, the small effect sizes underline that loneliness in geriatric patients is a complex and multifactorial phenomenon that cannot be explained by views on ageing alone. These findings support a biopsychosocial perspective on loneliness and suggest that subjective ageing may deserve greater consideration in geriatric assessment and future interventions. Longitudinal studies are needed to clarify the direction of these associations and to determine whether strengthening positive views on ageing can contribute to reducing loneliness in later life.

## Figures and Tables

**Table 1:** Sample characteristics.

Variable	N	Value
Age, years, mean ± SD	459	83.06 ± 5.85
Female, n (%)	459	301 (65.58)
Male, n (%)		158 (34.42)
Marital status, n (%)	458	
Single		31 (6.77)
Married/with partner		168 (36.68)
Widowed/separated		259 (56.55)
Missing		1 (0.22)
Living situation, n (%)	459	
Living alone		234 (50.98)
Living with partner/family		188 (40.96)
Other living arrangement		37 (8.06)
Education, n (%)	457	
Low education		15 (3.28)
Medium education		223 (48.80)
High education		219 (47.92)
Missing		2 (0.44)
Depressivity, Geriatric Depression Scale (GDS), mean ± SD	459	4.03 (2.97)
Barthel Index at admission to hospital, mean ± SD	459	46.78 (18.88)
VoA sum score, mean ± SD	459	13.21 ± 2.72
UCLA loneliness score, mean ± SD	459	1.54 ± 2.07
Felt age, years, mean ± SD	381	75.95 ± 11.64
Age from which a person is considered “old”, years, mean ± SD	296	77.21 ± 10.98

Note. The table describes participants with complete data on both the three-item UCLA Loneliness Scale and the Views on Ageing (VoA) sum score (N = 459). VoA = views on ageing; UCLA = three-item UCLA Loneliness Scale; SD = standard deviation. Higher UCLA scores indicate greater loneliness. Higher VoA scores indicate more negative views on ageing. Felt age was assessed with the item “Irrespective of your actual age, how old do you feel?” The perceived onset of old age was assessed with the item “Starting from which age would you consider someone ‘old’?”.

**Table 2:** Linear regression models predicting loneliness.

Model	Predictor	B	SE	β	*p*-Value	R^2^	Adjusted R^2^
Model 1	VoA sum score	0.12	0.04	0.16	<0.01	0.03	0.02
Model 2	VoA sum score	0.13	0.04	0.17	<0.01	0.03	0.02
	Age	−0.02	0.02	−0.05	0.26		
	Sex	−0.10	0.20	−0.02	0.61		
Model 3	VoA sum score	0.09	0.04	0.117	0.01	0.06	0.05
	Age	−0.02	0.02	−0.05	0.33		
	Sex	−0.10	0.20	−0.02	0.61		
	GDS	0.13	0.03	0.18	<0.01		

Note. Dependent variable: UCLA loneliness score. Model 1: unadjusted association between views on ageing and loneliness. Model 2: adjusted for age and sex. Model 3: additionally adjusted for depressive symptoms. Higher VoA scores indicate more negative views on ageing. UCLA = 3-item UCLA Loneliness Scale; VoA = views on ageing; GDS = Geriatric Depression Scale; B = unstandardized regression coefficient; SE = standard error; β = standardized regression coefficient.

**Table 3:** Fully adjusted regression models including subjective age measures.

Predictor	B	SE	β	*p*-Value	Interpretation
Felt age	0.02	0.01	0.11	0.04	Higher felt age predicted higher loneliness
Age threshold for being “old”	−0.03	0.01	−0.14	0.01	Later perceived onset of old age predicted lower loneliness

Note. Dependent variable: UCLA loneliness score. Each row represents a separate regression model. All models were adjusted for chronological age, sex, and depressive symptoms. UCLA = 3-item UCLA Loneliness Scale; B = unstandardized regression coefficient; SE = standard error; β = standardized regression coefficient.

## Data Availability

The datasets used during the current study are available from the corresponding author on reasonable request.

## Supplementary Materials

The following supporting information can be downloaded at: https://www.mdpi.com/article/10.3390/geriatrics11050125/s1. Supplementary Table S1. Bivariate associations between views on ageing and loneliness; Supplementary Table S2. Post-hoc group comparisons; Supplementary Table S3. Associations of depressive symptoms and covariates with loneliness and VoA.

## Abbreviations

The following abbreviations are used in this manuscript:

UCLA	University of California, Los Angeles
VoA	Views on Ageing
GDS	Geriatric Depression Score
DRKS	German Clinical Trials Register
OPS	Operations and Procedures Key System
DEAS	German Ageing Survey
