# Supplementary material for: Views on Ageing and Loneliness in Older Patients Undergoing Geriatric Treatment
Source: Geriatrics (Basel). 2026 Sep 7;11(5):125. doi: 10.3390/geriatrics11050125 (PMC13600205; doi:10.3390/geriatrics11050125)
Supplement: Supplementary file 1 [file geriatrics-11-00125-s001.zip › geriatrics-4512609-supplementary.pdf]

Supplementary Table S1. Bivariate associations between views on ageing and loneliness

Predictor	Association with loneliness	Spearman's ϱ / test result	p-value
VoA sum score	More negative VoA → higher loneliness	0.12	0.01
VoA 1: deterioration with age	No significant association	0.03	0.56
VoA 2: reduced vitality	More negative response → higher loneliness	0.15	0.01
VoA 3: reduced usefulness	No significant association	0.01	0.75
VoA 4: less favourable ageing expectations	More negative response → higher loneliness	0.13	0.01
VoA 5: lower happiness compared with younger age	More negative response → higher loneliness	0.15	0.01
Felt age	Higher felt age → higher loneliness	0.15	0.01
Age threshold for being "old"	Later threshold → lower loneliness	−0.14	0.02
Felt age group	Significant group difference	Kruskal-Wallis test	0.01

Note. Higher VoA scores indicate more negative views on ageing. Items 1 and 3 were recoded. VoA = views on ageing. Felt age was categorized relative to chronological age: 252 participants (61.60%) felt younger than their actual age, 103 (25.20%) felt the same age, and 54 (13.20%) felt older than their actual age.

Supplementary Table S2. Post-hoc group comparisons

Grouping variable	Outcome	Comparison	p-value	z-value
Felt age group	Loneliness	Feels same age vs. feels younger	0.03	2.54
Felt age group	Loneliness	Feels older vs. feels younger	0.01	3.14
Felt age group	Loneliness	Feels older vs. feels same age	0.83	1.09
Marital status	Loneliness	Single vs. married / with partner	0.01	3.11
Marital status	Loneliness	Single vs. separated / widowed	0.15	1.97
Marital status	Loneliness	married / with partner vs. separated / widowed	0.06	-2.32
Education	VoA sum score	High vs. low	0.04	-2.52
Education	VoA sum score	High vs. medium	1.00	0.86
Education	VoA sum score	Low vs. medium	0.01	2.85

Note. Post-hoc comparisons were conducted after significant or borderline group effects in non-parametric group comparisons. VoA = views on ageing. Higher VoA scores indicate more negative views on ageing. Felt age was categorized relative to chronological age: 252 participants (61.60%) felt younger than their actual age, 103 (25.20%) felt the same age, and 54 (13.20%) felt older than their actual age.

Supplementary Table S3. Associations of depressive symptoms and covariates with loneliness and VoA

Variable	Outcome	Association	Test statistic	p-value
Depressive symptoms (GDS)	Loneliness	Higher GDS → higher loneliness	ϱ = 0.16	<0.01
Depressive symptoms (GDS)	VoA sum score	Higher GDS → more negative VoA	ϱ = 0.27	<0.01
Depressive symptoms (GDS)	VoA 1	No significant association	ϱ = −0.02	0.65
Depressive symptoms (GDS)	VoA 2	Higher GDS → more negative response	ϱ = 0.24	<0.01
Depressive symptoms (GDS)	VoA 3	No significant association	ϱ = 0.03	0.52
Depressive symptoms (GDS)	VoA 4	Higher GDS → more negative response	ϱ = 0.23	<0.01
Depressive symptoms (GDS)	VoA 5	Higher GDS → more negative response	ϱ = 0.30	<0.01
Depressive symptoms (GDS)	Felt age	Higher GDS → higher felt age	ϱ = 0.24	<0.01
Depressive symptoms (GDS)	Age threshold for being "old"	No significant association	ϱ = −0.01	0.98
Chronological age	VoA sum score	Older age → more negative VoA	ϱ = 0.09	0.04
Chronological age	Loneliness	No significant association	ϱ = −0.04	0.44
Sex	VoA sum score	No significant association	ϱ = -0.01	0.95
Sex	Loneliness	No significant association	ϱ = −0.02	0.62
Marital status	Loneliness	Significant group difference	Kruskal-Wallis test	0.01
Marital status	VoA sum score	No significant group difference	Kruskal-Wallis test	0.61
Education	Loneliness	No significant group difference	Kruskal-Wallis test	0.06
Education	VoA sum score	Significant group difference	Kruskal-Wallis test	0.02
Living situation	Loneliness	No significant group difference	Kruskal-Wallis test	0.07
Living situation	VoA sum score	No significant group difference	Kruskal-Wallis test	0.21

Note. GDS = Geriatric Depression Scale; VoA = views on ageing; ϱ = Spearman's rho. Higher VoA scores indicate more negative views on ageing. Higher values for self-rated health indicate greater satisfaction with own health. Felt age was categorized relative to chronological age: 252 participants (61.60%) felt younger than their actual age, 103 (25.20%) felt the same age, and 54 (13.20%) felt older than their actual age.
